# Fluorimetric Mercury Test Strips with Suppressed “Coffee Stains” by a Bio-inspired Fabrication Strategy

**DOI:** 10.1038/srep36494

**Published:** 2016-11-04

**Authors:** Yuchun Qiao, Jizhen Shang, Shuying Li, Luping Feng, Yao Jiang, Zhiqiang Duan, Xiaoxia Lv, Chunxian Zhang, Tiantian Yao, Zhichao Dong, Yu Zhang, Hua Wang

**Affiliations:** 1 Shandong Province Key Laboratory of Life-Organic Analysis, College of Chemistry and Chemical Engineering, Qufu Normal University, Qufu, 273165, P. R. China

## Abstract

A fluorimetric Hg^2+^ test strip has been developed using a lotus-inspired fabrication method for suppressing the “coffee stains” toward the uniform distribution of probe materials through creating a hydrophobic drying pattern for fast solvent evaporation. The test strips were first loaded with the model probes of fluorescent gold-silver nanoclusters and then dried in vacuum on the hydrophobic pattern. On the one hand, here, the hydrophobic constraining forces from the lotus surface-like pattern could control the exterior transport of dispersed nanoclusters on strips leading to the minimized “coffee stains”. On the other hand, the vacuum-aided fast solvent evaporation could boost the interior Marangoni flow of probe materials on strips to expect the further improved probe distribution on strips. High aqueous stability and enhanced fluorescence of probes on test strips were realized by the hydrophilic treatment with amine-derivatized silicane. A test strips-based fluorimetry has thereby been developed for probing Hg^2+^ ions in wastewater, showing the detection performances comparable to the classic instrumental analysis ones. Such a facile and efficient fabrication route for the bio-inspired suppression of “coffee stains” on test strips may expand the scope of applications of test strips-based “point-of-care” analysis methods or detection devices in the biomedical and environmental fields.

Up to date, porous papers as the cost-effective and green flexible materials have been extensively applied for the fabrication of high-performance electronics^1^, electronic paper (e-Paper) technologies^2^, and test trips^3^,^4^,^5^. Particularly, the paper-based test strips, typically known as glucose test strip^3^, pregnancy diagnostic strip^4^, and urine test strip^5^, have been widely employed for diverse medial analysis, due to that they are cheap, readily use, and disposable for the in-site fast analysis. These papers have also been functionalized and especially combined with various microfluidic and diagnostic detection devices for the user-friendly detection alternatives to the conventional analytical instrumentations for “point-of-care” medical diagnosis, environmental monitoring, and food quality control^6^,^7^,^8^,^9^. For example, Lin’s group took the advantages of the chromatographic separation of test strips to develop the enzyme-linked immunoassays with electrochemical signal output for biomonitoring the exposures to insecticides^8^. Yu and co-workers developed an analytical device for photoelectrochemical immunoassay by using ZnO nanorod light-emitting diodes on flexible papers^9^. Nevertheless, most of the practical applications of test strips are still limited for the qualitative or half-quantitative analysis, since they have been being trapped by the undesirable distribution of the dispersed materials (i.e., probes) on test strips, so as to influence the outcome of the evaluation of signals and in turn the detection reliability and sensitivity^10^. Therefore, the distribution uniformity of the dispersed probe materials on test strips is considered as the formidable but attractive problem, which should be addressed before the test strips can be applied on a large scale.

It has been well established that the distribution uniformity of dispersed probe materials on test strips can be greatly challenged by the “coffee stains” resulting from the “coffee ring” effect, a formidable phenomenon that commonly takes place resulting in the deposition of material at the edge of evaporating droplets on a solid surface like a coffee droplet dry^11^,^12^,^13^. Despite many practical applications in printing^14^, biology^15^,^16^ and complex assembly^17^, such a ubiquitous nature of the “coffee stains” is factually difficult to avoid^18^,^19^,^20^,^21^,^22^,^23^, which may challenge the precision of printing and the quantitative performance of paper-based detection devices. Moreover, the “coffee stains” can occur in diverse systems with individual molecules^18^,^24^,^25^, bacteria^26^, and especially varying particle constituents from colloids to nanoparticles^11^,^18^,^24^,^27^. More seriously, unlike the commonly used nonporous solid substrates^11^,^19^,^28^, the “coffee stains” on the test strips of papers may become even more difficult to eliminate, since papers are porous material substrates in which the transport of the dispersed materials can be affected additionally by the chromatographic filtration effects and liquid evaporation^29^.

Over the past few decades, many efforts have been devoted to the suppression of “coffee stains”, but mostly focused on the non-porous solid substrates that are generally related to the occurrence of Marangoni effects^19^,^30^, including the introduction of gel^31^ or surfactants^26^,^32^, the change of deposition temperature^33^, the minimum of droplet size^34^, and the shape control^35^ or functionalization of dispersed materials^18^. For example, Yodh *et al*. reported that the shape of suspended particles, i.e., ellipsoidal particles, could help to counteract the “coffee stains”^35^. The surfactants have also been proved for depressing the “coffee stains” toward the homogeneous deposition of the bacteria on the solid plates upon drying^26^. Hegseth and co-workers established that under the fast evaporation condition the interior Marangoni flow of the droplets could be boosted so as to suppress the “coffee ring” effect^36^. Our group have also proposed the construction of hydrophobic surfaces to reverse the “coffee stains” to achieve the highly uniform and dense testing dots on the microarrays for the biomedical detections^37^,^38^. Moreover, up to date, a few investigations have been conducted on how to suppress the “coffee stains” on the porous substrates of test strips^28^,^29^, although this phenomenon has been generally ignored for the strips-based microfluidic and diagnostic analysis devices^8^,^39^,^40^,^41^,^42^,^43^,^44^. For example, Dou and co-workers studied the jet ink drops on the porous particle beds, confirming that the “coffee stains” on porous patterns could be influenced by the capillary draining and evaporation fluxes^28^. Zhang *et al*. developed a immunogold staining assay with nitrocellulose strips with the minimized “coffee stains”^45^. Remarkably, Shen’s group investigated the underlying mechanism of the “coffee stains” formation on the porous substrates like papers, demonstrating that the chromatographic filtration effects are generally the dominant driving forces for the control of particle transport in the porous paper substrates^29^,^46^.

It is recognized that the lotus plant can possess the outstanding ability to repel dirt by the superhydrophobicity or self-cleaning properties, known as the “lotus effect”^47^,^48^. This phenomenon has inspired a range of self-cleaning surfaces, antibacterial technologies, and especially fabrication strategies that may aid to control the microfluidic “lab-on-a-chip” devices^6^,^48^. In the present work, a lotus-inspired fabrication protocol has been proposed for the suppressing “coffee stains” on the test strips of porous papers by creating a hydrophobic pattern for drying the test strips in vacuum for fast reagent evaporation. As expected, the “coffee stains” could be dramatically minimized toward the uniform distribution of dispersed probe materials on test strips using gold-silver nanoclusters (Au-AgNCs) as the model probes for sensing Hg^2+^ ions in wastewater. To the best of our knowledge, this is the first attempt on the application of the “lotus effect” of superhydrophobicity and self-cleaning process for the suppression of “coffee stains” on test strips, in which the created hydrophobic drying pattern and the vacuum-aided fast reagent evaporation would control the exterior transport and boost the interior Marangoni flow of dispersed probe materials involved, respectively.

## Results

**Figure 1:**
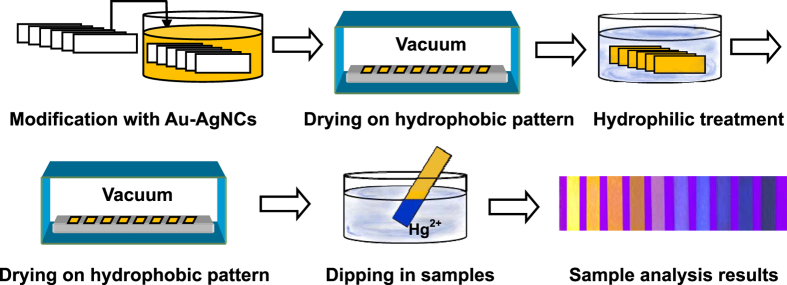
Schematic illustration of the fabrication and detection procedures of the Au-AgNCs-coated test strips.

It has been well established that the “coffee stains” can generally challenge the analysis performances of test strips by influencing the distribution uniformity of probes. Inspired by the “lotus effect” of superhydrophobicity and self-cleaning process^47^,^48^, herein, a “coffee stains”-suppressed fabrication protocol has been proposed for the fluorimetric test strips by creating a hydrophobic pattern for drying the probes-loaded test strips in vacuum. The main fabrication and detection procedures of fluorimetric test strips are schematically illustrated in Fig. 1. Here, the test strips were first loaded with the model probes of fluorescent Au-AgNCs^49^ and then dried in vacuum on a lotus surface-like hydrophobic pattern, which was created by coating the Hexadecyltrimethoxysilane (HDS) on the glass slides. As expected, the uniform distribution of dispersed fluorescent probes on test strips would be realized upon the suppressed “coffee stains”. Furthermore, the resulting test strips were treated by the hydrophilic aminopropyltriethoxysilane (APS) to be dried again on the HDS pattern in vacuum, so as to achieve the further improvement of the analysis performances of fluorimetric test strips such as the bettered surface hydrophilicity, enhanced probe fluorescence, and especially high environmental stability. Subsequently, the so prepared test strips were applied for the fluorimetric analysis of Hg^2+^ ions in wastewater samples by way of “dipping and drying”, where the Hg^2+^-triggered quenching of the fluorescence intensities of probes could occur.

**Figure 2:**
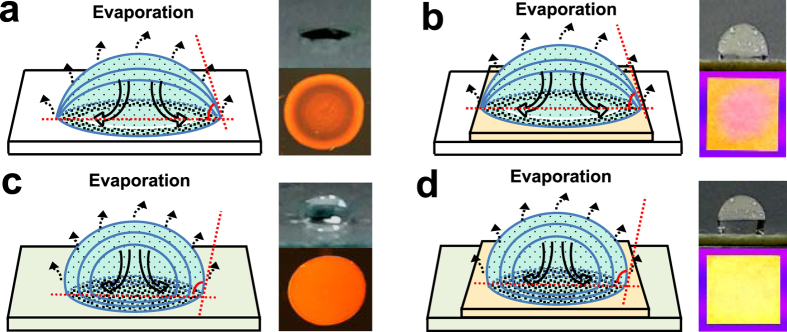
Schematics illustration of the main principle of the suppressed “coffee stains” of Au-AgNCs-containing droplets evaporated directly on the (**a**) bare and (**c**) hydrophobic HDS patterned glass slides, and the Au-AgNCs-loaded test strips dried on (**b**) the bare glass slide and (**d**) the hydrophobic HDS patterns, showing the dynamic moving procedures of Au-AgNCs (right) and the fluorescent photographs of the corresponding products (left).

Moreover, considering that the hydrophobic water-repellent double structure of the lotus surface (contact angles larger than 90°) can facilitate the significantly reduced contact area and the adhesion force between the surface and droplets leading to a self-cleaning process^47^,^48^, herein, the lotus-inspired underlying mechanism for suppressing “coffee stains” of the probe materials on test strips was explored, with the presumptive protocol schematically illustrated in Fig. 2. Here, the droplets with the dispersed probe materials were deposited separately on the glass slides with the hydrophilic (bare) surface and hydrophobic HDS-coated surface to comparably clarify two well-established evaporation modes involved: one at the constant contact area with the contact angle less than 90°, and another at the constant contact angle with the contact angle larger than 90°^50^,^51^. When a droplet of Au-AgNCs was deliberately evaporated on the hydrophilic surfaces of bare glass slides (contact angle of about 20°), the obtained contact area would keep constant but with the evaporation-induced diminishing contact angles (Fig. 2a). As a result, a fluorescent ring of Au-AgNCs would be left upon the solvent evaporation resulting from the “coffee ring” effect, as manifested in the microscopic image (right panel). However, when an Au-AgNCs-containing droplet was added onto the hydrophobic surface of HDS-modified slides (contact angle of about 127°), the constant contact angle with the evaporation-caused reducing contact area could be obtained so as to constrain the droplet within a spot (Fig. 2c), as also disclosed elsewhere^52^. Accordingly, the “coffee stains” on the hydrophobic glass surface would be largely minimized, so that a uniform fluorescent spot of Au-AgNCs was deposited upon drying. These evaporation and deposition behaviours of Au-AgNCs-containing droplets separately on the hydrophilic and hydrophobic solid glass slides above are also applicable for those on the porous test strips, which were dried separately on the bare glass slides (Fig. 2b) and the hydrophobic HDS pattern created alternatively (Fig. 2d). As schematically illustrated in Fig. 2b, Au-AgNCs could form the fluorescent “coffee stains” on test strips if dried on the hydrophilic substrate of bare glass slides. In contrast, they could be uniformly deposited on the test strips when dried on the lotus surface-like patterns of hydrophobic HDS showing the well suppressed “coffee stains”, as also visually illustrated in the corresponding photographs and microscopic images (Fig. 2d). Herein, the aforementioned hydrophobic constraining forces from the hydrophobic HDS patterns could be strong enough to balance the chromatographic or filtration forces that may otherwise control the transport of dispersed materials in porous papers^29^,^46^, so as to counteract the “coffee stains” towards a uniform deposition of dispersed probe materials on test strips. Moreover, when the Au-AgNCs-loaded test strips were dried in vacuum, a fast evaporation of solvent droplets would boost the interior Marangoni flow of probe materials involved^36^, thus leading to the further reduction of “coffee stains” on test strips. Additionally, the hydrophilic treatment for the test strips with amine-derivatized APS, which would be demonstrated afterward, would greatly improve the environmental stability, reagent penetration, and especially the fluorescence intensities of the fluorescent probes on test strips enhanced by the electronic donor effects.

**Figure 3:**
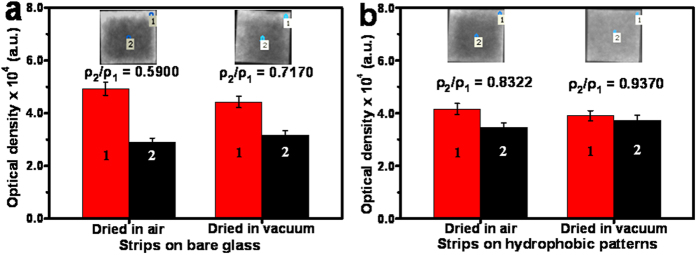
Comparison of the ρ_2_/ρ_1_ ratios of fluorescent Au-AgNCs between the test strips dried on (**a**) bare glass slide and (**b**) hydrophobic HDS pattern separately in air and vacuum, where the optical densities (ρ) of Au-AgNCs in (1) the edge areas and (2) the middle areas of test strips were measured. Insert: the calculated ρ_2_/ρ_1_ ratios and corresponding microscopic photographs.

Comparable investigations were comparably conducted on the distribution uniformities of the fluorescent probes of Au-AgNCs on test strips that were separately dried on the bare glass surface and the hydrophobic HDS pattern in vacuum or air (Fig. 3). Here, the probe distribution uniformities were monitored by calculating the ratios of the optical densities of Au-AgNC probes on test strips in ρ_2_/ρ_1_, where ρ_2_ and ρ_1_ refer to the average optical densities of probes in the middle and the edge areas of test strips, respectively^35^. As can be seen from Fig. 3a, the fluorescent Au-AgNCs could display the clear “coffee stains” on test strips when dried either in vacuum (right panel) or air (left panel) on the bare glass slides. Yet, the average probe ρ_2_/ρ_1_ ratio for the test strips dried in vacuum (0.7170) is a little bigger than that of the ones dried in air (0.5900), showing the relatively better suppression of “coffee stains” on test strips. Moreover, Fig. 3b manifests that the ρ_2_/ρ_1_ ratios were obtained separately for the strips dried in air (0.8322) and vacuum (0.9570), thus confirming that the “coffee stains” could be better suppressed for the test strips dried in vacuum on the hydrophobic HDS pattern. Importantly, the so created hydrophobic HDS patterns (Fig. 3b) could endow the fluorescent test strips much more uniform probe distribution than the bare glass ones (Fig. 3a) by comparison of their ρ_2_/ρ_1_ ratios. Herein, the fluorescent test strips could, on the one hand, benefit from the hydrophobic patterns (contact angle up to 127°) that could produce the lotus effect-like hydrophobic constrain forces to control the exterior transport of dispersed probe materials so as to minimize the “coffee stains” on test strips. On the other hand, the vacuum-aided fast solvent evaporation of test strips might help to boost the interior Marangoni flow of the solvent droplets involved^36^, so as to facilitate the further better distribution of fluorescent probes on test strips, thus promising the fluorimetric analysis with high sensitivity and reproducibility afterwards.

**Figure 4:**
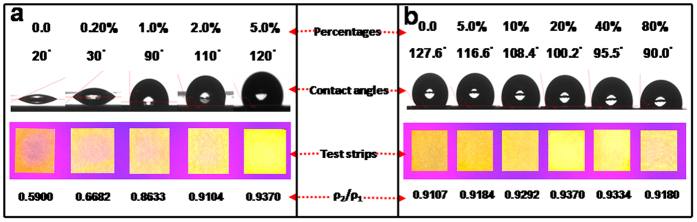
(**a**) The HDS dosages-dependent surface hydrophobicities of the patterns for the suppression of “coffee stains” on the test strips created with different HDS percentages. (**b**) The APS dosage-dependent surface hydrophilicities and fluorescent intensities of Au-AgNCs on test strips treated using APS of different percentages, showing different surface contact angles, the photographs of test strips under UV light, and the ratios of optical densities of Au-AgNCs in ρ_2_/ρ_1_.

It was found that the distribution uniformities of Au-AgNCs on test strips could depend on the hydrophobicities of the strip-drying patterns created with different HDS percentages (Fig. 4a). As described in Fig. 4a, the hydrophobicity degrees of the HDS patterns in contact angles could increase from 20° to 127° as the HDS percentages increased up to 5.0%, as confirmed elsewhere in our group^37^. More importantly, the ratios of optical densities (ρ_2_/ρ_1_) of Au-AgNCs on test strips could accordingly increase from 0.5900 to 0.9370 with the increasing hydrophobicities, as visually evidenced by the fluorescent photographs showing the different suppressions of “coffee stains” on test strips. Again, the hydrophobic constraining forces provided by the HDS patterns so created for drying the test strips might control the transport of the dispersed probe materials of Au-AgNCs on test strips by balancing the chromatographic or filtration forces of the porous substrates, so that the “coffee stains” could be significantly minimized toward the uniform distributions of the dispersed fluorescent probes on test strips.

The further hydrophilic treatments for Au-AgNCs-loaded test strips were investigated by using the amine-derivatized APS of different percentages (Fig. 4b). One can find that the surface hydrophilicities of test strips could increase with an increase in the APS percentages, reflected by the decreasing contact angles. Remarkably, the fluorescence intensities of Au-AgNCs on test strips could be dramatically enhanced when the APS percentages increased up to 20%, as witnessed in the fluorescent photographs of the test strips (Supplementary Fig. S4). Herein, the enhancement of the probe fluorescence intensities was thought to benefit from the electronic donor effects^38^, in which the amine groups of APS with protonic nitrogen atoms might serve as the electron donors for the fluorescent Au-AgNCs on test strips. However, higher APS percentages, i.e., 40% and 80%, were not attempted, since they could decrease the fluorescence intensities of the probes presumably resulting from the increasing aggregations of APS hydrolysis products. It could be substantially reflected by the corresponding ratios of optical densities (ρ_2_/ρ_1_) of Au-AgNCs on test strips that reached the maximum value of 0.9370 at the APS percentage of 20%, over which the worse distribution uniformities of Au-AgNCs on test strips could be encountered. Moreover, the hydrophilic treatment of APS (20%) could endow the faster penetration (within 10 s) of sample droplets into the yielded test strips (Supplementary Fig. S1c), in contrast to the one without APS treatment (Supplementary Fig. S1b). These data reveal that the APS treatment could improve the affinity of test strips to the sample solutions to achieve the rapid analysis. Also, the prepared Au-AgNCs-loaded test strips could obtain much higher environmental stability (up to six months) than the ones fabricated by the common way, which was defined as the common test strips (Supplementary Fig. S3a). Considerably high reproducibility of fluorimetry for sensing Hg^2+^ ions could thus be expected (Supplementary Fig. S3b), in addition to the active amine groups of test strips available for the further enrichment or binding of targeting ions (i.e., Hg^2+^ ions).

**Figure 5:**
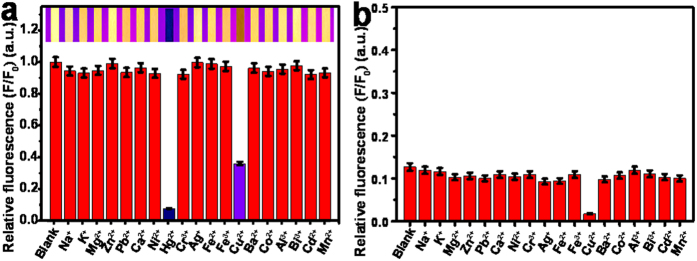
Visual fluorescent responses of the developed test strips to different metal ions with the images taken under UV light. (**a**) Fluorescence intensity changes of the developed test strips in the presence of different metal ions (3.0 μM) alone. (**b**) Fluorescence intensity changes of the developed test strips for Hg^2+^ ions separately co-existing other metal ions (3.0 μM) indicated.

To explore the sensing selectivity of the developed fluorimetric test strips, the visual fluorescent imaging was conducted comparably for probing 19 kinds of common metal ions (3.0 μM) (Fig. 5). One can note from Fig. 5 that the fluorescence of Au-AgNCs on test strips could be selectively quenched by Hg^2+^ ions. Herein, the relative fluorescence intensities of F/F_0_ were monitored, where F_0_ and F correspond to the fluorescence intensity of Au-AgNCs in the absence and presence of metal ions, respectively. Nevertheless, it should be pointed out that Cu^2+^ ions might also cause the fluorescence quenching of Au-AgNC probes to some degree. Herein, the possible interference from Cu^2+^ ions could be prevented by introducing the strong chelating agents of ethylenediaminetetraacetic acid (EDTA) with high enough concentration optimized as 5.0 μM, since the Cu^2+^-induced quenching mechanism might involve the interactions between Cu^2+^ ions and the protein scaffolds of Au-AgNCs by producing the protein−Cu^2+^complex^53^. In contrast, the fluorescence of Au-AgNCs could be quenched by Hg^2+^ ions through the formation of strong metallophilic Au−Hg^2+^ bonding, of which the lost fluorescence might not be recovered by EDTA^49^. In addition, the common anions might present no significant effect on the sensing selectivity of the developed fluorimetric test strips, as shown in Supplementary Fig. S5. Therefore, the developed fluorimetric test strips could allow for the visual analysis of Hg^2+^ ions with the high selectivity.

**Figure 6:**
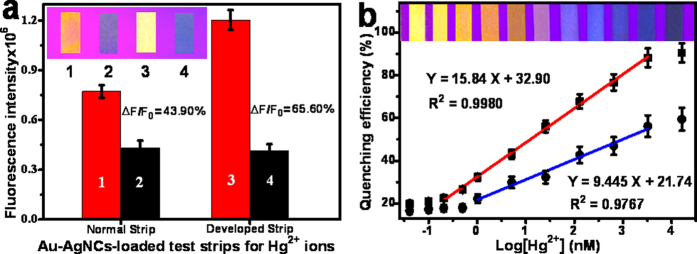
(**a**) Comparison of Hg^2+^-induced fluorescence responses between the common test strips and the developed test strips in the (1, 3) absence and (2, 4) presence of Hg^2+^ ions (0.10 μM), with the fluorescence quenching efficiencies. Insert: the corresponding photographs under UV light. (**b**) Fluorescence quenching efficiencies versus the logarithmic concentrations of Hg^2+^ ions spiked in wastewater analyzed by the developed test strips (red line) and the common test strips (blue line). Insert: the corresponding photographs of the developed test strips for Hg^2+^ ions under UV light.

The developed fluorimetry with the Au-AgNCs-loaded test strips was employed for probing Hg^2+^ ions in buffer by the “dipping and drying” way, in comparison to the common test strips (Fig. 6). Herein, the Hg^2+^-induced fluorescence quenching efficiencies of (F_0_−F)/F_0_ (defined as ΔF/F_0_) were calculated for the fluorescent probes on test strips. As can been seen from Fig. 6a that the developed test strips (65.60%) could exhibit the bigger fluorescence quenching efficiencies than the common ones (43.90%), presumably due to the partly Hg^2+^-inaccessible agglomeration and nonuniform distribution of Au-AgNCs on the common test strips resulting from the “coffee stains”. Accordingly, the developed test strips could present the higher probe quenching efficiencies to expect the higher sensing sensitivity to Hg^2+^ ions. Also, one can observe that the better uniformity distribution of the fluorescent probes on the developed test strips could be achieved resulting from the suppressed “coffee stains”, as shown in the photographs of corresponding products (Fig. 6a, insert).

Moreover, the feasibility of practical applications of the developed test strips was investigated by the fluorimetric analysis of Hg^2+^ ions spiked in wastewater (Fig. 6b) under the optimized detection conditions, i.e., 0.420 mM Au-AgNCs and pH 7.0 (Supplementary Fig. S6). It was discovered that the fluorescence quenching efficiencies increased as the Hg^2+^ concentrations increased, as apparently manifested in the photographs for the corresponding products (Fig. 6b, insert). A relationship between the quenching efficiencies and the logarithms of the concentrations of Hg^2+^ ions was obtained showing the linear Hg^2+^ concentrations ranging from 0.20 to 5000 nM (R^2^ = 0.9980), with a limit of detection of about 0.10 nM (Fig. 6b, red line). Furthermore, comparing to the previous analysis methods using the large and expensive instrumental detector (i.e., fluorescence spectrophotometer)^49^,^54^,^55^, the developed test strip method showed the comparable detection performance in terms of the detection limit and sensitivity. Yet, it could exhibit some outstanding advantages over most of the previously reported analysis methods such as the disposable strip, simple operation, and especially portable detector for promising for the “point-of-care” analysis and in-site monitoring of Hg^2+^ ions. Moreover, the comparative study was conducted for the Hg^2+^ detections using the common test strips (Fig. 6b, blue line), showing the linear Hg^2+^ concentrations from 1.0 to 5000 nM (R^2^ = 0.9767). Also, the detection limit of the developed test strip method is lower than that of other test strips-based Hg^2+^ analysis methods reported elsewhere^56^,^57^. These results indicate that higher detection sensitivity could be obtained by the developed test strips, in addition to the better reproducibility as demonstrated in Supplementary Fig. S3. Furthermore, some results obtained by the developed test strip-based fluorimetric method were compared to those of the classic ICP-MS method in the clinical laboratory (Supplementary Fig. S7). It was found that the regression equations for the different levels of Hg^2+^ ions were obtained with a correlation coefficient of 0.9923 (P > 0.050). Therefore, there is no significant difference in the analysis results between the two methods in quantifying Hg^2+^ ions in wastewater.

In summary, inspired by the “lotus effect” of superhydrophobicity and self-cleaning process, a novel fabrication strategy has been successfully developed to circumvent the formidable “coffee stains” on porous test strips through creating a hydrophobic pattern for drying the test strips in vacuum for fast solvent evaporation. The resulting dispersed fluorescent probes of Au-AgNCs could be deposited on test strips with greatly improved distribution uniformities for the fluorimetric analysis for Hg^2+^ ions in wastewater. The developed strips-based fluorimetry could possess some significant advantages over the common ones. First, the uniform deposition of sensing probes on test strips could be realized simply by suppressing the “coffee stains” through creating a hydrophobic drying pattern for the vacuum-aided fast solvent evaporation. Second, the further hydrophilic treatment of test strips with the amine-derivatized silicane could facilitate the high environmental stability and especially enhanced fluorescence intensities of the probes by the electronic donor effects, so as to expect the sensitive and reproducible detections. Third, a rapid penetration of sample solution into the test strips (within 10 s) could be realized allowing for the rapid fluorimetric analysis. Finally, the developed fluorimetric test strips could present the detection capacities comparable to the classic instrumental analysis methods in detecting Hg^2+^ ions. More importantly, such a lotus-inspired fabrication route for suppressing the “coffee stains” on disposable test strips may pave the way toward the extensive applications for the designs of diverse strips-based analysis methods and “point-of-care” detection devices with highly sensitive and reproducible analysis performances.

## Methods

### Materials and instruments

Tetrachloroauric acid (HAuCl_4_, 99.9%), silver nitrate (AgNO_3_), and bovine serum albumin (BSA) were purchased from Sigma-Aldrich (Beijing, China). Hexadecyltrimethoxysilane (HDS), aminopropyltriethoxysilane (APS), and ethylenediaminetetraacetic acid (EDTA) were bought from Sinopharm Chemical Reagent Co. (China). All of reagents we used are of analytical grade. Bimetallic alloying gold-silver nanoclusters (Au-AgNCs) were made in lab. Deionized water (>18 MΩ) was supplied from an Ultra-pure (UP) water system (Pall, USA). All glasses were thoroughly washed with aqua regia and rinsed with distilled water prior to use. Whatman filters papers cut for test strips were obtained from Sigma-Aldrich (Beijing, China). The wastewater samples from the local factory co-existing some other metal ions (i.e., Cr^3+^, Fe^3+^, Hg^2+^, and Pb^2+^) were spiked with Hg^2+^ ions of different concentrations with pH 6.5–7.4.

The step-by-step fabrication procedure of the test strips using silanization reagents and Au-AgNCs was monitored by the hydrophobic analysis of contact angles (CAs) using the Contact-angle measurement machine (Jinhe, Jiangsu, China). The optical densities for the characterization of the distribution of dispersed materials on the test strips were measured by Fluor Chem gel electrophoresis (FC2, Santa Clara, USA). The fluorescence measurements were conducted using fluorescence spectrophotometer (F-7000, Hitachi, Japan) operated at an excitation wavelength at 370 nm, with both excitation and emission slit widths of 5.0 nm. The fluorescence intensities were collected at 620 nm. The photographs of corresponding reaction products were taken with a digital camera under UV light with excitation wavelength at 365 nm. The wastewater samples were comparably analyzed in the clinical laboratory with an Inductively coupled plasma mass spectrometry (ICP-MS) of Agilent 7500ce (Agilent Technologies, Waldbronn, Germany).

### Synthesis of the fluorescent probes of Au-AgNCs

Red fluorescent Au-AgNCs were prepared according to the procedure reported previously in our group^49^. Briefly, HAuCl_4_ (10 mL, 10 mM) was added into BSA (10 mL, 50 mg mL^−1^). Then, AgNO_3_ (1.0 mL, 10 mM) was added to the mixture to be stirred for 5 min. Under vigorous stirring, NaOH (0.40 mL, 1.0 M) was added dropwise to be incubated for 12 h at 37 °C. Furthermore, the resulting Au-AgNCs were dialyzed in water for 48 h, and then collected to be stored at 4 °C for further use.

### Fabrication of the hydrophobic patterns of glass substrates

Flat and clear glass substrates (72 × 24 mm^2^) were cleaned by fresh piranha solution of H_2_SO_4_:H_2_O_2_ = 7:3 (Caution: piranha solution as a strong oxidant must be handled with extreme care) to activate the substrate surface, and then thoroughly washed in UP water to be further dried in nitrogen. Following that, those cleaned substrates were separately dipped into the hydrophobic HDS solutions of different percents in ethanol (0%, 0.10%, 1.0%, 2.0%, and 5.0%) to be reacted for 6 h at room temperature. After being washed for twice in ethanol and further dried, the surface contact angles of the resulting HDS-treated glass substrates were separately recorded. The so prepared hydrophobic glass patterns were kept in the sealing drier for future usage.

### Preparation of test strips of fluorescent Au-AgNCs

The preparation procedure of the fluorescent Au-AgNCs-loaded test strips for probing Hg^2+^ ions was schematically illustrated in Fig. 2. Typically, the filters papers were first cut into the slices of test strips (10 mm×10 mm) and then soaked into the above Au-AgNCs (0.420 mM) for 10 min. After that, the Au-AgNCs-loading test strips were immediately placed onto the hydrophobic HDS modified patterns to be dried in vacuum for 20 min or air overnight at room temperature. Furthermore, the resulting Au-AgNCs-loaded test strips were immerged separately into APS solutions of different percents in ethanol (0%, 5.0%, 10%, 20%, 40%, and 80%) for the functionally hydrophilic treatments for 30 s. Then, the APS-treated test strips were again placed onto the hydrophobic HDS modified patterns to be dried in vacuum for 10 min at room temperature. Moreover, the common test strips of Au-AgNCs for the control tests were fabricated accordingly, except for use of the normal glass slides as the drying substrates for the test strips, where all of the dried test strips were stored in dark for future usage. Subsequently, the so obtained fluorescent Au-AgNCs-loaded test strips were separately applied for the paper-based detection of Hg^2+^ ions simply by the “dipping and drying” way. Meanwhile, the calibration curve of the test strips-based fluorimetry for Hg^2+^ samples was obtained by using different Hg^2+^ concentrations in wastewater (0.20, 1.0, 5.0, 25, 125, 625, 1250, and 5000 nM), in which 5.0 μM EDTA was introduced to avoid any interference of potentially co-existing Cu^2+^ ions. The analysis results obtained by the developed test strips were compared to those of the common fluorimetric test strips and the classic ICP-MS.

## Additional Information

**How to cite this article**: Qiao, Y. *et al*. Fluorimetric Mercury Test Strips with Suppressed “Coffee Stains” by a Bio-inspired Fabrication Strategy. *Sci. Rep.* **6**, 36494; doi: 10.1038/srep36494 (2016).

**Publisher’s note:** Springer Nature remains neutral with regard to jurisdictional claims in published maps and institutional affiliations.

## Supplementary Material

Supplementary Information
