# Supplementary material for: Fluorimetric Mercury Test Strips with Suppressed “Coffee Stains” by a Bio-inspired Fabrication Strategy
Source: Sci Rep. 2016 Nov 4;6:36494. doi: 10.1038/srep36494 (PMC5095603; doi:10.1038/srep36494)
Supplement: Supplementary Information [file srep36494-s1.pdf]

Supplementary Information for

Fluorimetric Mercury Test Strips with Suppressed “Coffee Stains” by a Bio-inspired Fabrication Strategy

Yuchun Qiao, Jizhen Shang, Shuying Li, Luping Feng, Yao Jiang, Zhiqiang Duan, Xiaoxia Lv, Chunxian Zhang, Tiantian Yao, Zhichao Dong, Yu Zhang & Hua Wang*

Shandong Province Key Laboratory of Life-Organic Analysis, College of Chemistry and Chemical Engineering, Qufu Normal University, Qufu, 273165, P. R. China.

E-mail addresses: huawangqfnu@126.com; Tel: +86 537 4456306; Web: http://wang.qfnu.edu.cn.

List of Contents

Figure S1. The photographs of time-dependent penetrations of Hg^{2+} sample droplets into the Au-AgNCs-loaded test strips before and after the hydrophilic APS treatment.

Figure S2. The microscopic images for the distributions of Au-AgNCs dropped on the glass slides without the hydrophobic HDS pattern or vacuum drying after the APS treatments.

Figure S3. Comparison of environmental stabilities and Hg^{2+}-sensing reproducibility between the Au-AgNCs-loaded test strips fabricated by the common procedure and the developed route.

Figure S4. Comparison of the fluorescent intensities between the Au-AgNCs-loaded test strips before and after the treatment of amine-derivatized APS for six repetitive detections.

Figure S5. Fluorescent intensity changes of the developed test strips in sensing different anions alone.

Figure S6. Optimization of main detection conditions of the test strips-based fluorimetry including Au-AgNCs concentrations and pHs.

Figure S7. The correlation of the detection results for analyzing Hg^{2+} ions obtained by the developed strips-based fluorimetry and the classic ICP-MS method.

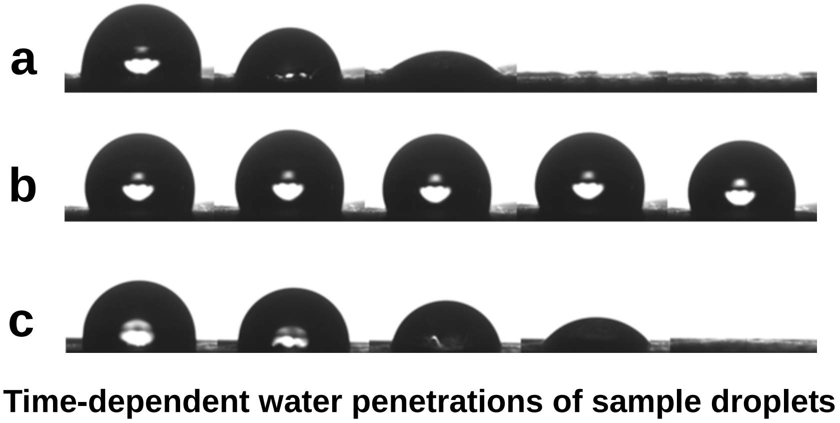

Figure S1. The photographs of time-dependent penetrations of Hg^{2+} sample droplets into the Au-AgNCs-loaded test strips (**b**) before and (**c**) after the hydrophilic APS treatment, with (**a**) the blank strip (without loading of Au-AgNCs) as the control, of which the photographs of the sample droplets were taken at time intervals of 2 s under the conditions: 0.420 mM Au-AgNCs, 5.0 % HDS, 20 % APS, and drying time of 10 min at room temperature.

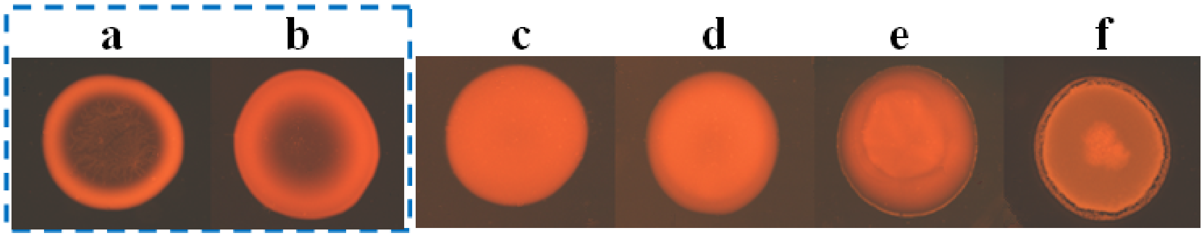

Figure S2. The microscopic images for the distributions of Au-AgNCs dropped on the glass slides (**a**) without the pre-coating of the hydrophobic HDS and (**b**) without the drying in vacuum; (**c**)-(**f**) the distributions of Au-AgNCs dropped on the glass slides coated with the hydrophobic HDS pattern and dried in vacuum, followed by the further treatment with APS of different percentages (10 %, 20 %, 40 %, and 80 %), respectively, under the conditions: 0.420 mM Au-AgNCs, 5.0 % HDS, and drying time of 10 min at room temperature.

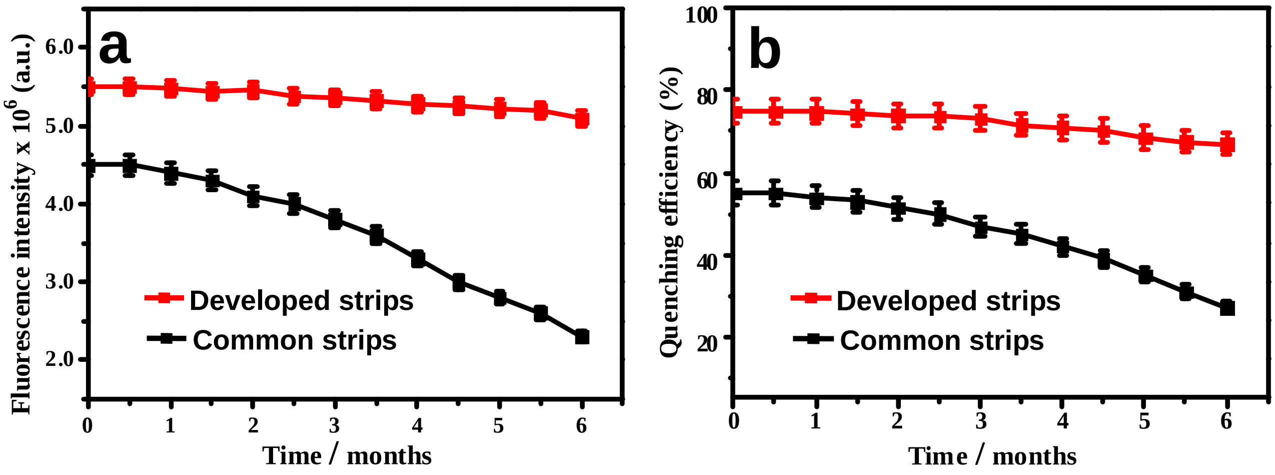

Figure S3. Comparison of (**a**) environmental stabilities and (**b**) Hg^{2+}-sensing reproducibility between the Au-AgNCs-loaded test strips fabricated by the common procedure (known as the common test strips) and the developed route, where the fluorescent intensities and Hg^{2+} quenching efficiencies were comparably measured under the conditions: 0.420 mM Au-AgNCs, drying time of 10 min at room temperature, and the test strips stored away over different time intervals.

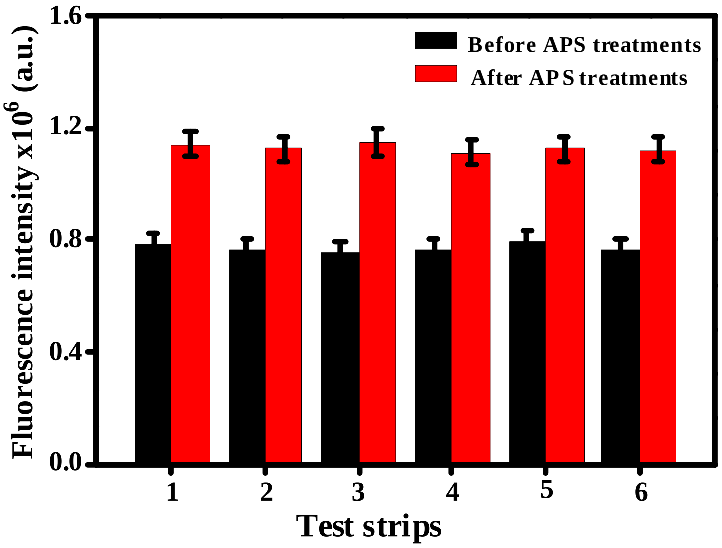

Figure S4. Comparison of the fluorescent intensities between the Au-AgNCs-loaded test strips before and after the treatment of amine-derivatized APS for six repetitive detections under the conditions: 0.420 mM Au-AgNCs, 5.0 % HDS, 20 % APS, drying time of 10 min at room temperature, where the test strips were dried on the hydrophobic HDS patterns in vacuum.

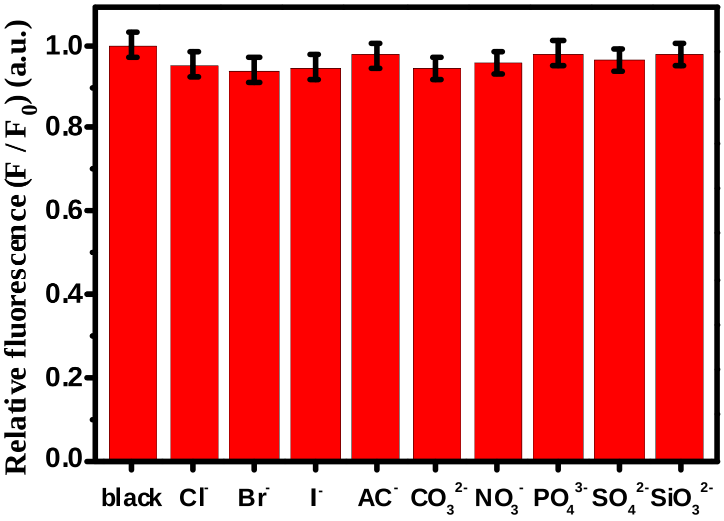

Figure S5. Fluorescent intensity changes of the developed test strips in sensing different anions (3.0 μM) alone under the conditions: 0.420 mM Au-AgNCs, 5.0 % HDS, 20 % APS, drying time of 10 min at room temperature.

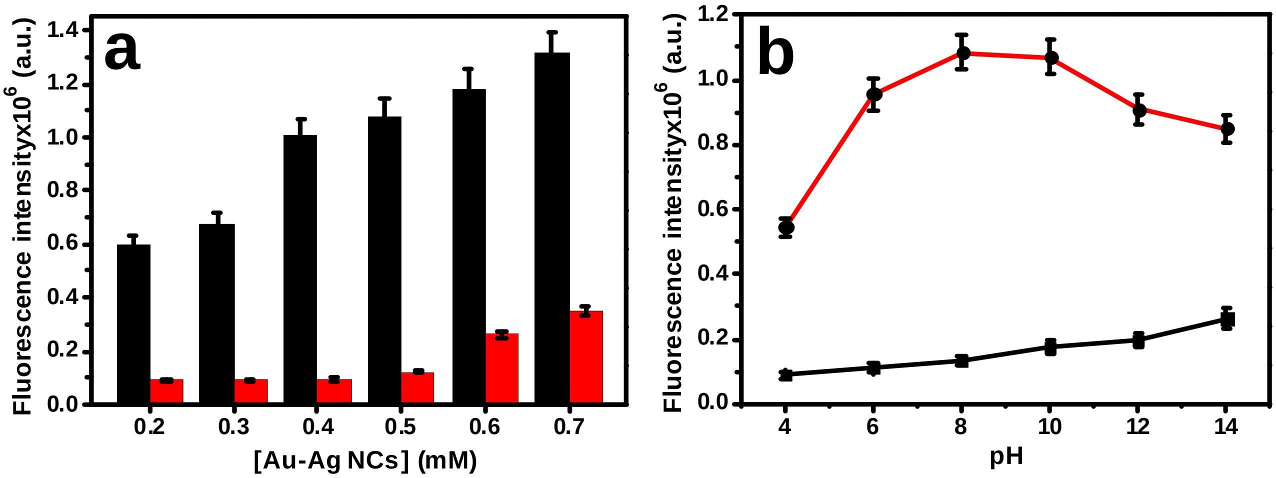

Figure S6. Optimization of main detection conditions of the test strips-based fluorimetry including (a) Au-AgNCs concentration-dependent fluorescence intensities and (b) the pH-dependent fluorescence intensities in the absence (red line) and presence (black line) of Hg^{2+} ions under the conditions: 5.0 % HDS, 20 % APS, drying time of 10 min at room temperature.

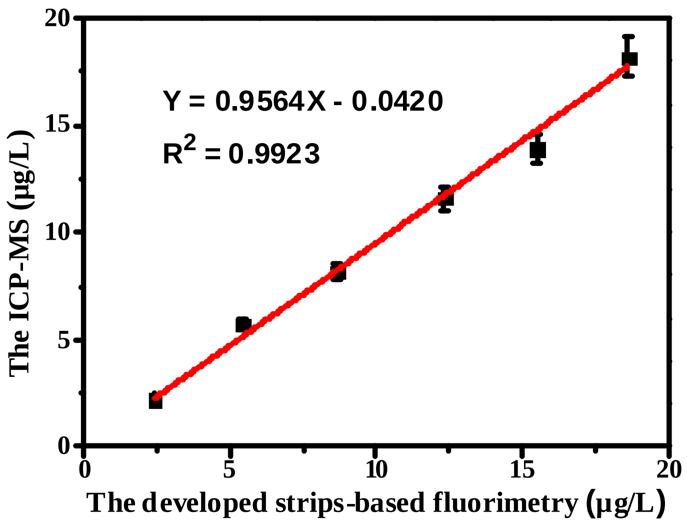

Figure S7. The correlation of the detection results for analyzing Hg^{2+} ions obtained by the developed strips-based fluorimetry and the classic ICP-MS method under the conditions: 0.420 mM Au-AgNCs, 5.0 % HDS, 20 % APS, drying time of 10 min at room temperature, and Hg^{2+} ions with different levels in wastewater.
